# Controllable Electrochemical Synthesis of Reduced Graphene Oxide Thin-Film Constructed as Efficient Photoanode in Dye-Sensitized Solar Cells

**DOI:** 10.3390/ma9020069

**Published:** 2016-01-25

**Authors:** Soon Weng Chong, Chin Wei Lai, Sharifah Bee Abd Hamid

**Affiliations:** Nanotechnology & Catalysis Research Centre (Nanocat), Institute of Postgraduate Studies (IPS), University of Malaya, 3rd Floor, Block A, 50603 Kuala Lumpur, Malaysia; soonweng86@gmail.com (S.W.C.); sharifahbee@um.edu.my (S.B.A.H.)

**Keywords:** reduced graphene oxide, electrochemical reduction, voltages, dye-sensitized solar cells (DSSCs), efficiency

## Abstract

A controllable electrochemical synthesis to convert reduced graphene oxide (rGO) from graphite flakes was introduced and investigated in detail. Electrochemical reduction was used to prepare rGO because of its cost effectiveness, environmental friendliness, and ability to produce rGO thin films in industrial scale. This study aimed to determine the optimum applied potential for the electrochemical reduction. An applied voltage of 15 V successfully formed a uniformly coated rGO thin film, which significantly promoted effective electron transfer within dye-sensitized solar cells (DSSCs). Thus, DSSC performance improved. However, rGO thin films formed in voltages below or exceeding 15 V resulted in poor DSSC performance. This behavior was due to poor electron transfer within the rGO thin films caused by poor uniformity. These results revealed that DSSC constructed using 15 V rGO thin film exhibited high efficiency (η = 1.5211%) attributed to its higher surface uniformity than other samples. The addition of natural lemon juice (pH ~ 2.3) to the electrolyte accelerated the deposition and strengthened the adhesion of rGO thin film onto fluorine-doped tin oxide (FTO) glasses.

## 1. Introduction

The rapid growth of the global population and industrial activities pose a great challenge to meeting ever-increasing energy demands in the future. Currently, world electricity generation is dominated by fossil fuels such as coal, oil, and natural gas. However, fossil fuels are non-renewable resources that might be depleted, which simultaneously causes the prices of these fuels to rise over the years. Nevertheless, the combustion of fossil fuels emits greenhouse gases such as carbon dioxide that greatly impact global warming and climate change. Electricity is the fastest growing form of energy, and net global electricity generation is projected to grow by 2.2% annually from 2008 to 2035 [1]. Thus, the high demand of energy supply has motivated researchers to seek alternative sources of energy.

Renewable energy sources such as solar, hydro, wind, and biomass show great potential in contributing to electricity generation. Among these sources, solar energy is the most abundant and sustainable and has therefore become a potential candidate as an energy source. Numerous studies have been conducted on photoconversion systems such as silicon-based solar cells and dye-sensitized solar cells (DSSCs). DSSC conversion efficiency might be less than the silicon-based thin-film cells, but its low price/performance ratio allows it to compete with fossil fuel electrical generation by achieving grid parity. The Gratzel cell, an early version of a DSSC, was first introduced in 1988 by Brian O’Regan and Michael Gratzel at UC Berkeley. The DSSC is a low-cost thin-film solar cell that consists of semiconductors formed between a photosensitized anode and an electrolyte that work together as a photoelectrochemical system. The DSSC offers numerous attractive features such as low cost and simple fabrication, good stability, and compatibility with flexible substrates [2,3,4]. The DSSC is basically a low-cost thin-film solar cell that consists of semiconductors formed between a photo-sensitized anode and an electrolyte that work together as a photoelectrochemical system [5,6,7,8]. The DSSC offers numerous attractive features such as it being easy to make, and having low cost fabrication cost, good stability and compatibility with flexible substrates [2,9,10]. However, the technology faces a few remaining hurdles before large-scale commercial production can realize that promise.

Some recent works have proven that graphene possesses great properties such as optical transparency, high stability, low cost, and non-toxicity, making it a promising alternative photoanode material for DSSC [11,12]. The most attractive property of graphene oxide (GO) is that it can be partly reduced to graphene-like sheets by removing the oxygen-containing groups with the recovery of a conjugated structure. The reduced graphene oxide (rGO) sheets are usually considered one kind of chemically derived graphene. The most straightforward goal of any reduction protocol is to produce graphene-like materials similar to the pristine graphene. Numerous efforts have been exerted by researchers in this matter, and several reduction strategies were introduced such as thermal reduction, chemical reduction, electrochemical reduction, and multi-step reduction [13]. Chemical reduction is a commonly used strategy to reduce GO into rGO. Unfortunately, this method generally utilizes reducing agents that are toxic or explosive such as hydrazine and sodium borohydride [14]. As a result, continuous efforts have been directed towards the exploration of an eco-friendly reducing method for GO reduction. To address this issue, the electrochemical reduction of GO is an alternative that relies on the removal of oxygen functionalities [15,16,17,18]. This method can be carried out in a normal electrochemical cell using an aqueous buffer solution at room temperature.

For the above matter, Zhou *et al.* [15] reported the best reduction effect using an electrochemical method. Elemental analysis of the resultant rGO revealed a C/O ratio of 23.9, and the conductivity of the rGO film produced was measured at approximately 85 S/cm. They found that the potential needed to realize the reduction is controlled by the pH value of the buffer solution. A low pH value is favorable to the reduction of GO. Thus, the authors proposed that H^+^ ions participate in the reaction. Meanwhile, An *et al.* [17] used electrophoretic deposition (EPD) to make GO films. They suggested that GO sheets can also be reduced on the anode surface during EPD, which seems counterintuitive to the general belief that oxidation occurs at the anode in an electrolytic cell. Although the reduction mechanism is unclear, the simultaneous film assembly and reduction might be favorable to some electrochemical applications. Therefore, in this work, we have fabricated the rGO thin film as a photoanode in a DSSC by using the electrochemical reduction method with the addition of lemon juice as the buffer solution to investigate its performance. The project aims to attain high efficiency of DSSC by controlling the uniformity of the rGO thin film. The effect of the potential applied toward the formation of rGO is addressed. Fine-tuning the potential applied in this process is important to develop an efficient and cheap method for producing rGO thin films.

## 2. Methodology

### 2.1. Graphene Oxide (GO) Synthesis

**Figure 1:**
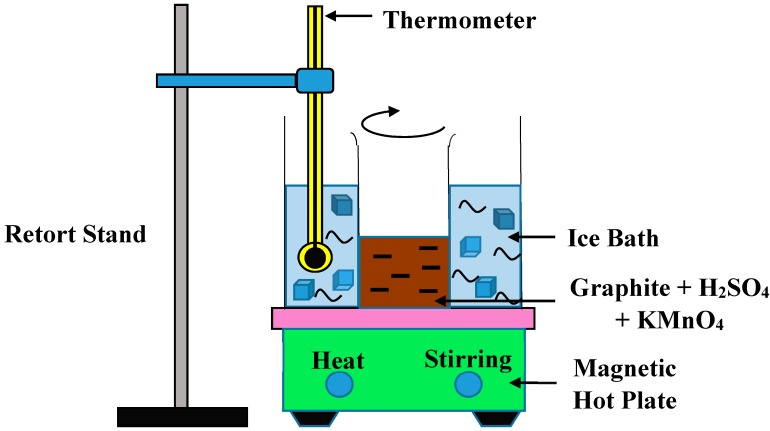
Experimental setup of simplified Hummer’s method.

In the present study, GO was synthesized by using the simplified hummers method [19]. In this method, 3 g of graphite (graphite flakes, Sigma Aldrich, St. Louis, MO, USA) was mixed with 70 mL of sulfuric acid (H_2_SO_4_) (0.5 M, Chemolab, Seri Kembangan, Selangor, Malaysia) in the ice bath environment. During stirring at a constant speed, 9 g of potassium permanganate (KMnO_4_) (Chemolab) was added very slowly into the mixture. At this stage, the temperature of the suspension was kept below 20 °C to prevent any possible explosion from the exothermic reaction. The temperature was raised to 35 °C and stirred for 30 min after the KMnO_4_ was added completely. Next, 150 mL of deionized water was added and the temperature was raised to 95 °C. To stop the reaction, 500 mL of water and 15 mL of hydrogen peroxide (H_2_O_2_) (30%, Chemolab) were added into the suspension. Then, the suspension was washed with 10 mL hydrochloric acid (HCl) (1M, Chemolab) and then centrifuged at 7000 rpm for 15 min. The experimental setup was shown in Figure 1. The supernatant was decanted, and the sediment was washed with deionized water and brought to centrifugation again. This washing process was performed to remove metal ions [20]. The washing process was repeated twice and then dried in the oven at 90 °C for 24 h.

### 2.2. Electrochemical Reduction

**Figure 2:**
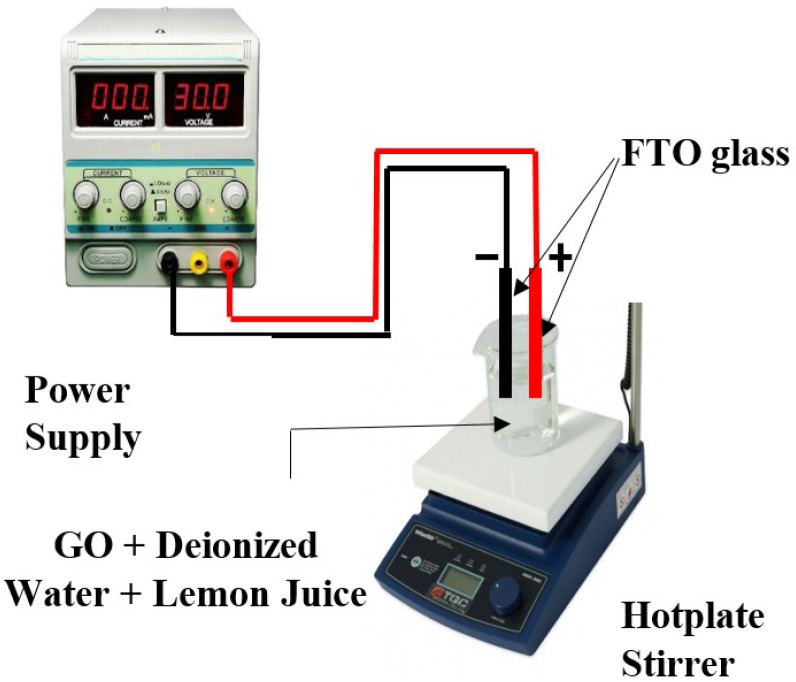
Experimental setup of electrochemical-reduction method.

Fluorine Doped Tin Oxide coated glass slide (FTO, Sigma Aldrich) was coated with the rGO samples by using the electrochemical reduction method. The FTO glasses (2 cm^2^ each) were immersed into a mixture of 100 mL of deionized water (DI) water, 5 mL lemon juice and 0.01 g of GO powder. The lemon juice was extracted from unripe lemon fruit by squeezing out the juice and then sifted with a stainless steel sifter. The lemon juice was tested with a pH meter and showed a pH of approximately 2.3. This process was performed on a set of different voltages; 5, 10, 15, 20, and 25 V for 5 min. The instrument setup was as shown in Figure 2.

### 2.3. Preparation of DSSC

The FTO glasses coated with rGO had an active area of approximately 1 cm^2^ each. The FTO glasses were heated in the furnace at 450 °C for 15 min. After that, the FTO glasses were soaked into solution containing anhydrous ethanol and 0.5 mM N-719 dye for 24 h. Then, these dye-sensitized rGO films were rinsed with acetonitrile in order to eliminate the physisorbed N-719 dye molecules. Subsequently, the reference electrodes were prepared by burning the conductive side of the FTO glass on candle. The rGO-coated photoanodes were then sandwiched together with the reference electrodes by using paper clips. A few drops of KI electrolyte (0.5 M) were then carefully applied between the photoanodes and reference electrodes. The excess electrolytes were removed by using clean wipes.

### 2.4. Characterization

**Figure 3:**
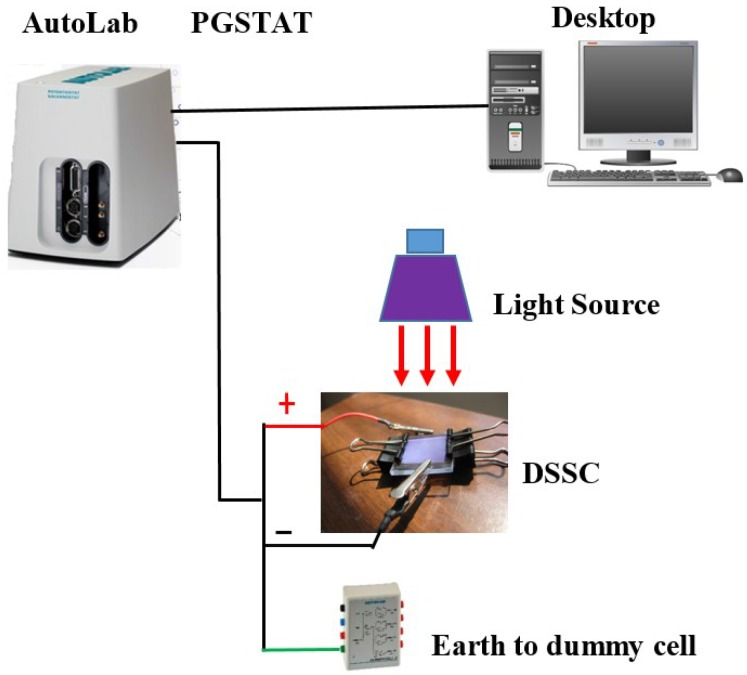
Electrical characterization setup using AutoLab PGSTAT204 system.

The changes in functional groups were determined by using Fourier transform infrared spectroscopy (FT-IR) (Bruker-IFS 66/S, Bruker Corporation, Billerica, MA, USA). The scan was conducted from 500 to 4000 cm^−1^. The phase determination of the GO and rGO was determined by X-ray diffraction (XRD) using a D8 Advance X-Ray Diffractometer (Bruker AXS, Bruker Corporation) at a scanning rate of 0.033°·s^−^^1^, 2Ɵ from 2° to 90° with CuKα radiation (λ = 1.5418 Å). The vibrational and rotational modes as well as the crystallinity of the samples were investigated by using the Raman spectroscopy (Renishaw inVia Microscope, HeCd laser, Renishaw plc, Gloucestershire, UK). The surface morphologies of GO and rGO were observed by field emission scanning electron microscopy (FESEM, FEI, Hillsboro, OR, USA), using a FEI Quanta 200F Environmental SEM at 5.0 kV and a working distance of 10 mm. The thickness and surface roughness were characterized using an atomic force microscope (AFM; Bruker Multimode 8 Instruments, Bruker Corporation), and data were analyzed by using Nanoscope Analysis software (Metrohm, Petaling Jaya, Selangor, Malaysia). Meanwhile, for the electrical characterization, the FTO glasses coated with the rGO samples were constructed into DSSCs and connected to the AutoLab PGSTAT204 instrument (Metrohm) to examine its electrical performance as shown in Figure 3. Linear sweep Voltammetry was performed with a voltage sweep from 0 to 0.7 V.

## 3. Results and Discussion

**Figure 4:**
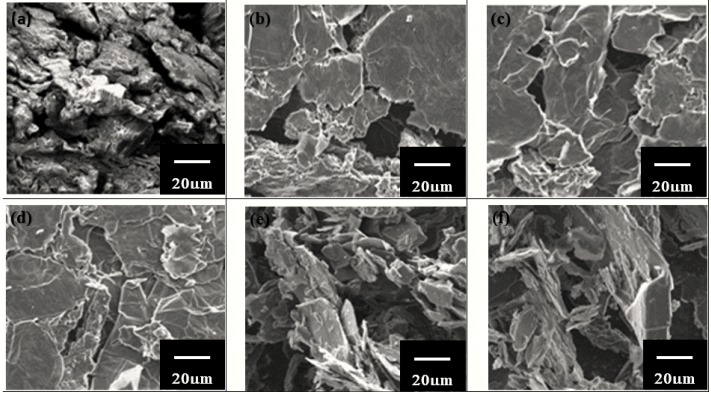
FESEM images of (**a**) GO; GO electrochemically reduced at (**b**) 5 V, (**c**) 10 V, (**d**) 15 V, (**e**) 20 V, and (**f**) 25 V.

For the morphological analysis, the rGO thin-film samples were examined under the FESEM. The FESEM produces better resolution because of the intensive and monochromatic electronic beam employed. Figure 4 shows the FESEM images of (a) GO; GO electrochemically reduced at (b) 5 V, (c) 10 V, (d) 15 V, (e) 20 V, and (f) 25 V. These images were taken at a magnification of 3k. As observed from Figure 4, the 15 V rGO thin-film sample had the best uniformity as compared with the other samples. The 5 V and 10 V samples were quite uniformly coated with rGO, but were not as well coated the 15 V sample was. The samples coated at 20 V and 25 V, which showed bumpy and coarse surfaces, decreased the efficiency of the DSSC produced because of reduced surface area for dye adhesion. These images indicate that the 15 V rGO thin-film sample was reduced more effectively as compared with the other samples. This assumption was confirmed by the FTIR, XRD, and Raman results. The elements present in the rGO thin films are shown in Table 1.

**Figure 5:**
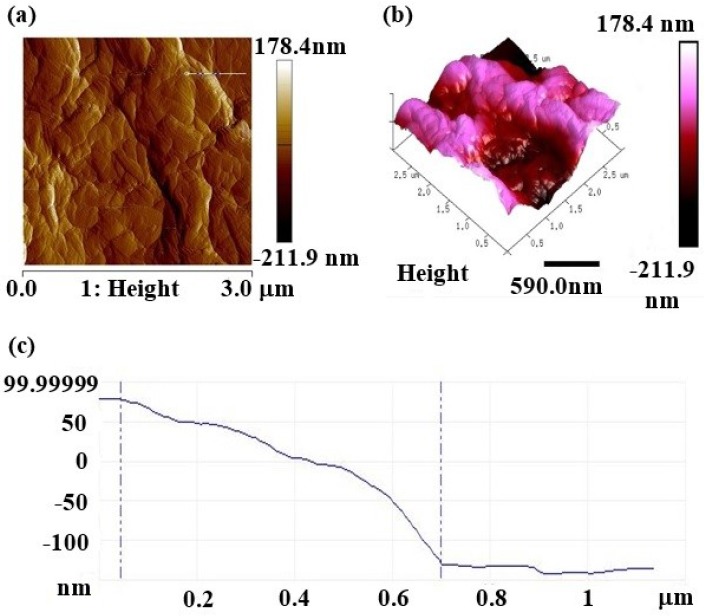
AFM topology of (**a**) 2D height, (**b**) 3D height, and (**c**) line profile of 15 V rGO thin film sample.

The Atomic Force Microscopy (AFM) was used to study the surface topology and height profiles of rGO [21]. The two-dimensional (2D), three-dimensional (3D) and height profile of the 15 V rGO thin-film sample were shown in Figure 5. The light pink color in the 3D image represented the highest point of the sample surface, whereas the dark red regions represented the valley or sample pores [22]. The height profile of our 15 V rGO thin-film sample showed that the thickness achieved through the electrochemical reduction for 5 min was 207.8 nm. The thickness of this sample was found to be more chemically stable as a thinner layer of the film would have easily peeled off from the FTO glass under harsh chemicals such as strong acid/base solutions, although the thickness could be controlled by manipulating the duration of the reduction process. The addition of lemon juice, which contained a combination of citric acid and ascorbic acid during the electrochemical reduction, had introduced more surface adsorptive sites (mainly carboxylic functional groups) onto the rGO to provide greater adhesion on the FTO glass [23]. However, the surface roughness, *R_a_* of this sample was recorded at a value of 12.11 nm. The thickness, roughness, and surface roughness for all the rGO thin films are listed in Table 2.

**Table 1:** Energy-dispersive X-ray spectroscopy (EDX) analysis of elements present in reduced graphene oxide (rGO) thin films prepared at 5, 10, 15, 20, and 25 V by electrochemical reduction for 5 min.

Materials	Carbon (%)	Oxygen (%)	Sulphur (%)	Chlorine (%)	Sodium (%)	Total (%)
rGO thin-film (5 V)	76.20	23.00	0.08	0.29	0.43	100
rGO thin-film (10 V)	80.60	18.57	0.09	0.28	0.46	100
rGO thin-film (15 V)	84.32	15.06	0.07	0.20	0.35	100
rGO thin-film (20 V)	75.21	24.02	0.08	0.22	0.47	100
rGO thin-film (25 V)	73.17	26.19	0.07	0.25	0.32	100

**Table 2:** Surface analysis of rGO thin-films prepared at 5, 10, 15, 20, and 25 V by electrochemical reduction for 5 min.

Sample	Roughness Parameters		
	Thickness (Mean), nm	Roughness (*R*_max_), nm	Surface Roughness (*R*_a_), nm
rGO thin-film (5 V)	178.2	19.021	10.23
rGO thin-film (10 V)	189.3	19.876	11.78
rGO thin-film (15 V)	207.8	21.095	12.11
rGO thin-film (20 V)	224.7	24.571	12.54
rGO thin-film (25 V)	256.8	28.922	13.42

**Figure 6:**
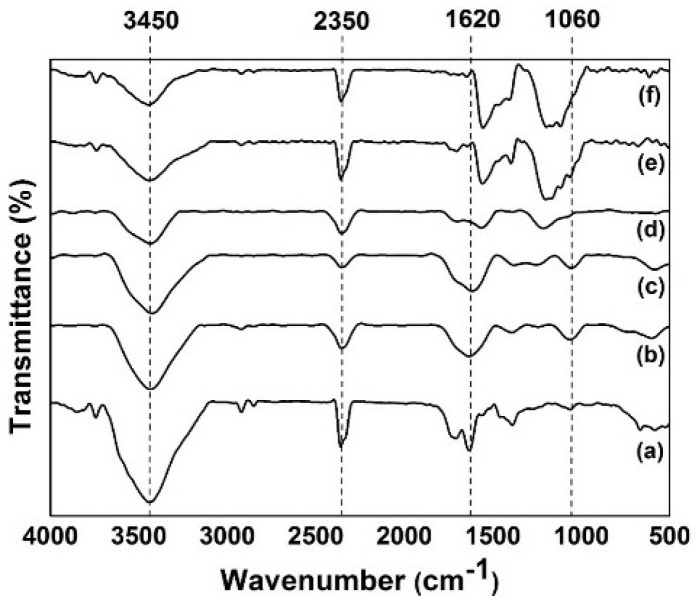
FT-IR spectra of (**a**) GO; GO electrochemically reduced at (**b**) 5 V, (**c**) 10 V, (**d**) 15 V, (**e**) 20 V, and (**f**) 25 V.

The FT-IR spectra were used to determine the functional groups in our samples. Figure 6 shows the FT-IR spectra of a set of rGO thin-film samples deposited on FTO glasses with varying voltages: 5, 10, 15, 20, and 25 V. The electric field induced reduction [24,25] of the GO film took place in the presence of our natural reducing agent (lemon juice). Under an applied voltage, the GO is directed toward our targeted electrode. During the process, the ionization of interlayer water molecules in the GO suspension yields hydrogen ions and hydroxyl ions because of the strong electric field [26]. The reduction of GO occurred as in Equation (1) [25].

(1) GO + 2H^+^ + 2e^−^ → rGO + H_2_O

The high intensity of the main peaks in GO (Figure 6a) confirms the presence of a large amount of oxygen functional groups after the oxidation process [19]. The graphs show the stretching of the hydroxyl group at 3450 cm^−1^ [27] and carbon dioxide at 2350 cm^−1^ [19]. The peak at 1620 cm^−1^ in Figure 6a–d shows the presence of the alkene group (C=C) [19] where this peak shifted to 1590 cm^−1^ in Figure 6e,f which might be attributed to the enhancement in the aromatic region is due to π–π stacking [28]. The peak at 1415 cm^−1^ can be assigned to a C–O bond (carboxyl functional group). The peak at 1220 cm^−1^ is because of C–OH (epoxy functional group). The peak at 1060 cm^−1^ suggests a C–O bond (alkoxy functional group) [29,30]. As observed from Figure 6d, the GO functional groups significantly decreased when 15 V was applied in this electrochemical reduction process. This decrement in the intensity shows that the GO was reduced effectively.

This reduction process was accelerated because of the added lemon juice, which has reducing property and provides additional hydrogen ions. In addition, the lemon juice has a pH of 2.3, provides acid ascorbic, which promotes a strong acidic environment to effectively eliminate oxygen functional groups on carbon planes [31,32]. As a result, the deposited GO film on the targeted FTO glass was simultaneously reduced. In addition, the FTIR spectra suggest that the reduction process exerted the best effect in an applied voltage of 15 V as compared with the other applied voltages because of the oxygen functional groups decreased significantly [33,34]. The deposited film samples in voltages exceeding 15 V showed a reverse effect, which reverted the rGO film back to GO film. This phenomenon may be due to the restacking of GO sheets by van der Waals forces after the deposited rGO film became saturated. However, the amorphous nature of rGO, the complexity of chemical reactions, and the lack of means to directly monitor the reduction process have hindered the elucidation of the exact reduction mechanism.

**Figure 7:**
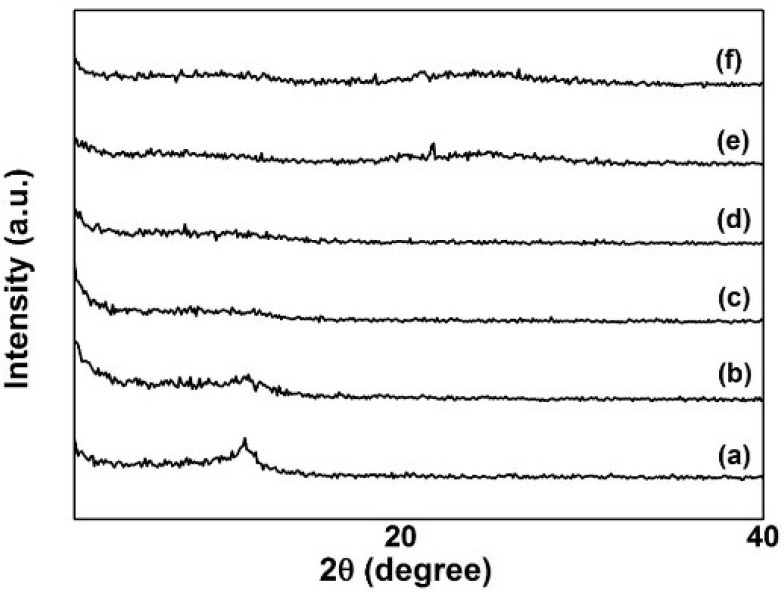
XRD spectra of (**a**) GO; GO electrochemically reduced at (**b**) 5 V, (**c**) 10 V, (**d**) 15 V, (**e**) 20 V, and (**f**) 25 V.

In this study, XRD analysis was used to determine the crystal structure, the orientation and the interlayer distance between GO and rGO. Our previous work reported that the pristine graphite employed a sharp and high intensity diffraction peak at 2θ = 26.7°, which describes a highly organized layer structure with an interlayer distance (d-spacing) of 0.34 nm along the (002) orientation [19]. After the oxidation process, the (002) peak is shifted to 2θ = 10.9° (Figure 7a) giving an increase in the d-spacing from 0.34 to 0.81 nm, suggesting that single- or several-layered GO nanosheets were prepared. This result was confirmed by the AFM data. This increase in d-spacing was inflicted by the intercalation of oxygen functional groups and water molecules into the graphite interlayers [35,36].

In this work, the GO was electrochemically reduced in five different voltages, as shown in Figure 7. The peak of 2θ = 10.9° disappeared after being reduced at Figure 7 (b) 5 V, (c) 10 V, (d) 15 V, (e) 20 V, and (f) 25 V. The decrement in the intensity of the peak clearly indicates that the oxygen containing groups of the GO have been efficiently removed [37]. However, a small peak at 2θ = 23° started to appear after 15 V (Figure 7e,f), indicating a decrease in the interlayer spacing. The reasons for this phenomenon may be due to the loosely stacked GO nanosheets being drawn closer to the adjacent ones by a strong van der Waals interaction [38]. These obtained XRD spectra (Figure 7e,f) further confirmed the FT-IR spectra in the previous section.

The difference in the structure of GO and rGO lies in a large amount of chemical functional groups attached to the carbon plane and structural defects within the plane, both of which can severely decrease electrical conductivity. As a result, the reduction of GO can be considered to aim at achieving the elimination of functional groups. The conductivity of monolayer graphene mainly relies on carrier transport within the carbon plane. As a result, functional groups attached to the plane are the main influencing factor on its conductivity, whereas functional groups attached to the edge have less influence. Consequently, the reduction of GO must be mainly aimed at eliminating epoxy and hydroxyl groups on the plane, while other groups, for example, carboxyl, carbonyl and ester groups, presenting at the edges or defective areas, only have a limited influence on the conductivity of an rGO sheet [39].

**Figure 8:**
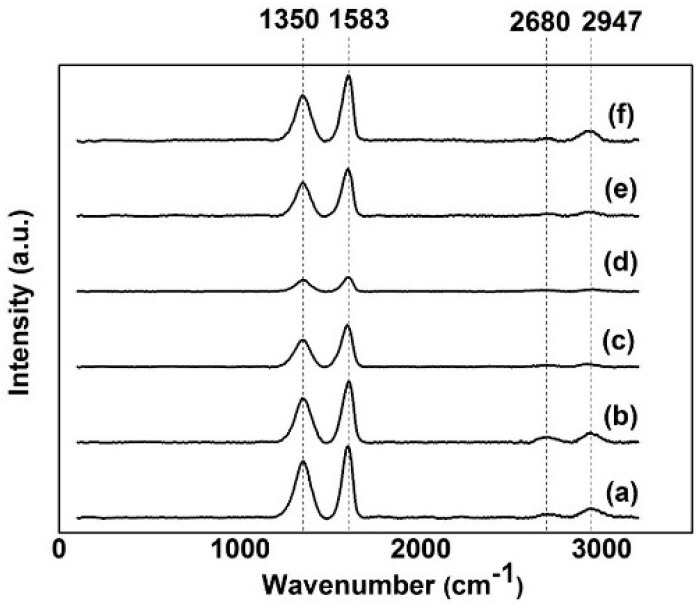
Raman spectra of (**a**) GO; GO electrochemically reduced at (**b**) 5 V, (**c**) 10 V, (**d**) 15 V, (**e**) 20 V, and (**f**) 25 V.

The Raman spectroscopy is very useful for characterizing sp^2^ and sp^3^ hybridized carbon materials such as graphite, fullerenes, carbon nanotubes, and graphene. The Raman fingerprints can be used to differentiate single, double, and multi-layer graphenes. Raman spectroscopy utilized a monochromatic laser to interact with molecular vibrational modes and phonons in a sample, shifting the laser energy up or down through inelastic scattering [40]. The 514 nm excitation laser was occupied in this Raman analysis. As observed from Figure 8, the D-mode, appears at approximately 1350 cm^−1^, and the G-mode appears at approximately 1583 cm^−1^. These two main peaks are the second-order overtone of a different in-plane vibration, as well as the primary in-plane vibrational mode [41]. The G-mode arises from the stretching of the C–C bond in graphitic materials, and is common to all sp^2^ carbon systems. The G-band is highly sensitive to strain effects in sp^2^ system and thus can be used to probe modification on the flat surface of graphene. The D-mode is caused by a disordered structure of graphene. The first-order D peak cannot be visible in pristine graphene because of crystal symmetries [42]. For the D peak to occur, a charge carrier has to be excited and inelastically scattered by a phonon, and then a second elastic scattering by a defect or zone boundary must occur to result in recombination [43]. The presence of disorder in sp^2^-hybridized carbon systems results in resonance Raman spectra, and thus makes Raman spectroscopy one of the most sensitive techniques to characterize disorder in sp^2^ carbon materials.

For the GO samples that were electrochemically reduced within 15 V, two other modes exist at 2680 cm^−1^ (2D-mode) and 2947 cm^−1^ (D+G-mode). As the number of graphene layers increases, the spectrum changes from that of a single layer graphene (2D peak) into an increasing number of modes that can combine to give a wider, shorter and higher frequency peak because of the added force from the interactions between layers of stacked graphene [44]. Thus, this electrochemical reduction technique produced multi-layered rGO on the FTO glasses. Through calculation, the ratio of *I*_D_/*I*_G_ for GO was 0.54 whereas rGO that was electrochemically reduced at 15 V was 0.79. This increase indicates that a change in the electronic conjugation state of GO has occurred during reduction [45,46]. Given that the *I*_D_/*I*_G_ intensity ratio is inversely proportional to the average size of the sp^2^ domains, the higher *I*_D_/*I*_G_ ratio for rGO indicates that rGO is smaller than GO in plane sp^2^ domains [45,47].

**Figure 9:**
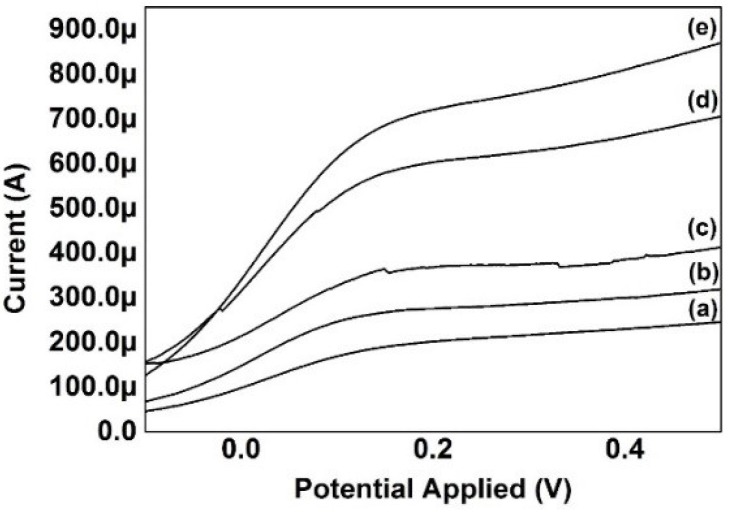
Current–voltage curves generated by AutoLab system for GO electrochemically reduced at (**a**) 25 V, (**b**) 20 V, (**c**) 5 V, (**d**) 10 V, and (**e**) 15 V.

The rGO thin-films conductances were investigated by using the AutoLab system at a voltage sweep from −0.1 V to 0.5 V. The surface area of the FTO glass was 1 cm^2^. Figure 9 illustrates the calculated current–voltage curves of the rGO thin-films and the conductance were tabulated in Table 3. The calculations were based on Equations (2) and (3):

(2) $$\text{Resistance},\;R=\frac{V}{I}$$

(3) $$\text{Conductance},\;G=\frac{1}{R}=\frac{I}{V}$$

A C atom contains six electrons, two in the inner shell and four in the outer shell. The outer shell electrons are responsible for chemical bonding. However, each atom in graphene is connected to three other C atoms on the two dimensional plane, leaving one free electron for electronic conduction [19]. These high-mobility free electrons located above and below the graphene sheets, called the pi (π) electrons, are responsible for the calculated value of the conductance. As observed from the calculated values of Table 3, the conductance of the rGO thin-film reduced at 15 V was significantly higher in comparison to the rGO thin films reduced at other voltages. This phenomenon may be attributed to the lower content of oxygen in the rGO reduced at 15 V as compared with the other samples. Thus, more electrons were able to travel within the rGO reduced at 15 V, providing a higher current output. As discussed in the FT-IR section, the reappearing of the oxygen functional groups when the voltage exceeded 15 V has caused the resistance to be higher. The thin-film samples reduced at voltages exceeding 15 V exhibits the behavior of the GO instead of rGO.

**Table 3:** Resistance and conductance values of rGO thin-films prepared at 5, 10, 15, 20, and 25 V by electrochemical reduction for 5 min calculated at voltage, *V* = 0.5 V.

Materials	Voltage (V)	Current (μA)	Resistance (Ω)	Conductance (μ Ω^−1^)
rGO thin-film (5 V)	0.5	413	1210.7	826.0
rGO thin-film (10 V)	0.5	705	709.2	1410.0
rGO thin-film (15 V)	0.5	869	575.4	1737.9
rGO thin-film (20 V)	0.5	318	1572.3	636.0
rGO thin-film (25 V)	0.5	245	2040.8	490.0

**Figure 10:**
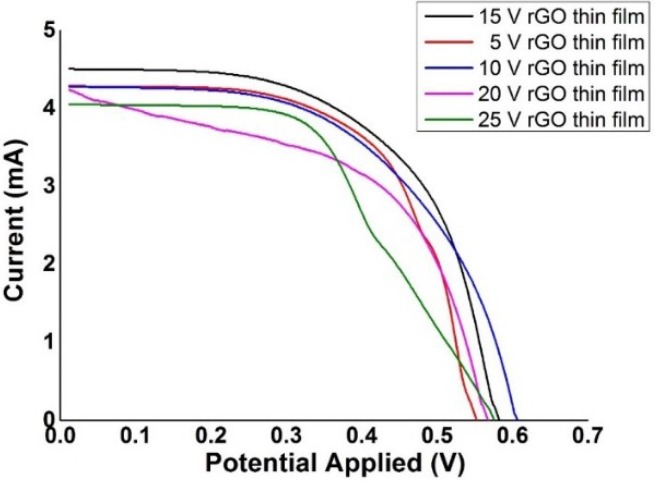
DSSC performance of rGO thin-film photoanode produced from electrochemical reduction in various voltages.

To investigate the electrical performance, the rGO thin-films were constructed into photoanodes of DSSCs and tested by using AutoLab PGSTAT204. Each of the 5, 10, 15, 20, and 25 V rGO thin-film DSSCs were swept with voltage from 0 to 0.7 V. The dimension of the photovoltaic (PV) cells was fixed at 1 cm^2^, whereas the light source has a power of 100 W. The current-voltage characteristics obtained from the AutoLab PGSTAT204 system were plotted in Figure 10.

The fill factor and efficiency were calculated based on Equations (4) and (5) and the results were tabulated in Table 4:

(4) $$FF=\frac{I_{\max}\times V_{\max}}{I_{\mathrm{SC}}\times V_{\mathrm{OC}}}$$

(5) $$\eta =\frac{I_{\max}\times V_{\max}}{P_{\text{light}}}=\frac{FF\times I_{\mathrm{SC}}\times V_{\mathrm{OC}}}{P_{\text{light}}}$$

**Table 4:** *V*_OC_, *I*_SC_, *FF*, and η for DSSC calculated from Figure 10.

Electrochemical Reduction Applied Voltage, (V)	Open-Circuit Voltage, *V*_OC_ (V)	Short-Circuit Current, *I*_SC_ (A)	Maximum Voltage, *V*_max_ (V)	Maximum Current, *I*_max_ (A)	Fill Factor, *FF*	Efficiency, η (%)
5	0.553	4.29	0.46	2.81	0.5449	1.2927
10	0.607	4.28	0.48	2.78	0.5136	1.3413
15	0.583	4.51	0.41	3.71	0.5785	1.5211
20	0.568	4.24	0.48	2.39	0.4763	1.1471
25	0.576	4.05	0.38	3.01	0.4903	1.1438

The DSSC constructed by using the 15 V rGO thin-film was found to have higher efficiency than that of the other samples. This result is in agreement with the FESEM results because the 15 V sample has the most uniformly coated surface among the other samples. However, the efficiency achieved by rGO alone was still low because the rGO thin-film was incorporated as the photoanode instead of the reference electrode. The photoconversion efficiency could possibly be improved by the incorporation of metal oxides such as TiO_2_.

## 4. Conclusions

In summary, a fast and simple method to obtain rGO thin film by using lemon juice in the electrochemical reduction was demonstrated. In this work, the best voltage for electrochemical reduction was around 15 V, proven by the higher photoconversion efficiency in DSSCs. Using a voltage lower than 15 V showed an inadequate degree of uniformity of the deposited rGO thin film, whereas when the voltage applied exceeded 15 V, the rGO thin film became rough. The uniformity of the rGO thin film directly affected the efficiency of the DSSC devices. Moreover, the use of lemon juice accelerated the reduction and improved the adhesion of the thin film onto FTO glasses. In addition, this natural and environmentally friendly reducing agent is cheap and is highly available.
